# The movement of mitochondria in breast cancer: internal motility and intercellular transfer of mitochondria

**DOI:** 10.1007/s10585-024-10269-3

**Published:** 2024-03-15

**Authors:** Sarah Libring, Emily D. Berestesky, Cynthia A. Reinhart-King

**Affiliations:** Department of Biomedical Engineering, Vanderbilt University, 440 Engineering and Science Building, 1212 25thAvenue South, Nashville, TN 37235 USA

**Keywords:** Breast cancer, Mitochondria, mtDNA, Tunneling nanotubes, Mitochondrial transfer

## Abstract

As a major energy source for cells, mitochondria are involved in cell growth and proliferation, as well as migration, cell fate decisions, and many other aspects of cellular function. Once thought to be irreparably defective, mitochondrial function in cancer cells has found renewed interest, from suggested potential clinical biomarkers to mitochondria-targeting therapies. Here, we will focus on the effect of mitochondria movement on breast cancer progression. Mitochondria move both within the cell, such as to localize to areas of high energetic need, and between cells, where cells within the stroma have been shown to donate their mitochondria to breast cancer cells via multiple methods including tunneling nanotubes. The donation of mitochondria has been seen to increase the aggressiveness and chemoresistance of breast cancer cells, which has increased recent efforts to uncover the mechanisms of mitochondrial transfer. As metabolism and energetics are gaining attention as clinical targets, a better understanding of mitochondrial function and implications in cancer are required for developing effective, targeted therapeutics for cancer patients.

## Introduction

Breast cancer is the most commonly diagnosed cancer globally, with approximately 2.3 million new cases and 685,000 deaths in women in 2020 [1]. 5-year overall survival is > 80% if diagnosed at stage I or II (American Joint Commission on Cancer Staging, 6th edition), but was 63.4% for individuals diagnosed at stage III and 22.8% at stage IV using the 2006–2010 Surveillance, Epidemiology, and End Results (SEER) data [2]. In 2011, Hanahan and Weinberg expanded the hallmarks of cancer to include reprogramming cellular metabolism. Cancer cells have long been known to prefer glycolysis over oxidative phosphorylation (OXPHOS) even in the presence of oxygen (i.e. aerobic glycolysis), which is termed “the Warburg Effect” and has been successfully utilized in clinical scans for decades [3, 4]. Aerobic glycolysis has been associated with *c-MYC* and *RAS* amplifications and with loss of *TP53*, while increased glycolysis in general can also be attributed to hyperplasia and the hypoxic cores associated with advanced tumors [3, 5]. As such, metabolic reprogramming varies across breast cancer molecular subtype depending on the most common oncogenic drivers and phenotypic presentation of each. For example, triple negative breast cancer cells have the highest expression of GLUT1 (glucose transporter 1) and display the most dramatic switch from mitochondrial respiration to glycolytic energy production across the molecular subtypes [5]. First proposed by Warburg himself, the prevailing hypothesis was that aerobic glycolysis in tumor cells occurred due to irreversible defects in the mitochondria. However, advances in the last 25 years have revealed that many cancer cells still retain the capacity for OXPHOS during disease progression [6, 7]. For example, glycolysis inhibitors tested did not have as significant of an effect on tumor growth as expected. Additionally, increased OXPHOS is often seen in cancer stem cells and cancer cells resistant to chemotherapy [8]. These observations suggest that tumors are metabolically heterogeneous and that at least a subset of cancer cells are likely metabolically plastic. More recently, mitochondria have also been shown to actively participate in several cell fate decisions, such as cell cycle control and programmed cell death control. Altogether, mitochondria and mitochondrial metabolism have found renewed interest in basic research and for clinical targeting [6, 9, 10]. Here we will discuss an overview of the transfer of mitochondria and mitochondria-related content in cancerous tissues, with particular emphasis on breast cancer.

## Mitochondrial basics

### Mitochondrial structures

**Fig. 1:**
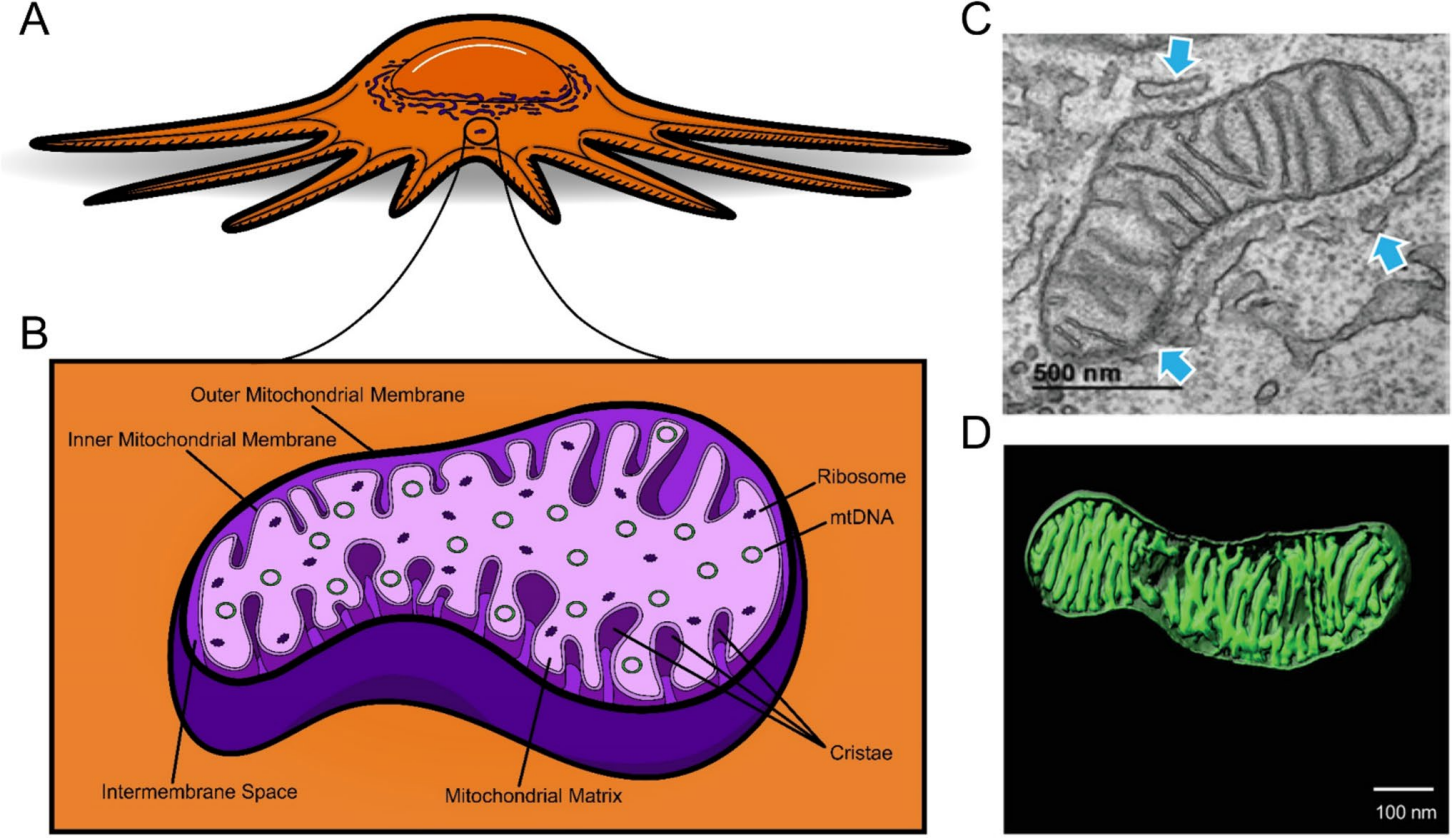
Mitochondrial structure. Graphical representations of **A** mitochondria in a cell with **B** key structures inside a mitochondrion labeled. **C** Transmission electron microscope (TEM) image of a mitochondrion from control skeletal muscle myotubes. Blue arrows indicate areas of mitochondria-endoplasmic reticulum contact as quantified in the source work [19] **D** 3D reconstruction of cristae morphology in wildtype mouse retina using a focused ion beam scanning electron microscope. **C** and **D** adapted from Hinton et al. [19] under CC 4.0

Mitochondria are energy-producing organelles that form interconnected network(s) within the cytoplasm of a cell (Fig. 1A) [11]. These organelles are tubular, membrane-bound structures, approximately 0.5–3 µm in length. In fact, a mitochondrion has a double-membrane, consisting of an inner and outer mitochondrial membrane separated by intermembrane space (Fig. 1B) [11, 12]. The inner membrane invaginates into the inner most compartment of the mitochondria (the mitochondrial matrix), creating folds known as cristae (Fig. 1B–D). Voltage-dependent anion channels located on the outer mitochondrial membrane connect the cytosol of the cell to the intermembrane space and allow small molecules such as ions and nucleotides to flow throughout the mitochondrion [13]. These channels and other ion channels help to establish a membrane potential across the outer and inner mitochondrial membranes. This potential is essential for the generation of ATP via the tricarboxylic (TCA) cycle and the electron transport chain (ETC), which occur in the matrix and at the inner mitochondrial membrane, respectively [14]. Each crista can act as an individual unit and is connected to the inner boundary membrane through a narrow tubular junction that is believed to limit diffusion of OXPHOS-related molecules [15]. As such, cristae within one mitochondrion may have disparate membrane potentials (which are separate from the inner boundary membrane potential as well). Crista structure is dynamic, fluctuating between a contracted/dense state and a wide/less dense state to adapt to the demands of the mitochondrion’s environment [15–17]. Given the role of mitochondrial membrane potential in ATP generation, it is often used as a marker of overall mitochondrial activity [18].

### Mitochondrial DNA (mtDNA)

Mitochondria uniquely have their own, maternally-inherited genome, present in multiple copies in each mitochondrion [20]. Mitochondrial DNA (mtDNA) is housed in the mitochondrial matrix, along with the mitochondrial ribosomes and the intermediates and byproducts of the TCA cycle. Human mtDNA is 16,569 nucleotides long and encodes 37 genes for 22 tRNAs, 2 rRNAs, and 13 proteins involved in the ETC, as well as a control region [21–23]. The genetic material is stored in the form of nucleoids, which are closed circular molecules [24]. The rest of the mitochondrial proteome, composed of approximately 1500 proteins, is encoded in the DNA in the nucleus of the cell (nDNA) [20]. Present in at least hundreds of copies per cell and being transcribed continuously, human mtDNA accumulates mutations at a rate at least an order of magnitude greater than nDNA [20, 25, 26]. Frequently, more than one mtDNA variant is present within the same cell, known as heteroplasmy [27]. To combat the effects of a mutant allele overtaking a population, mtDNA switching is possible both inter- and intracellularly. Intercellular exchange, or horizontal transfer, of mtDNA will be discussed in detail below (“The flow of mitochondria from surrounding cells in breast cancer”). Intracellularly, mitochondria can disperse and mix their genetic contents by fusing membranes together to become larger, elongated mitochondria in a process known as fusion [22, 28]. During this process, old or non-functional mitochondria are repurposed by cross-complementation and mixing of mitochondrial contents [28]. These large mitochondria can then disseminate into multiple independent mitochondria via fission. Fusion and fission are two complementary processes—mitofusin 1 and 2 (Mfn1 and Mfn2) and optic atrophy-1 (OPA1) fuse the outer and inner membranes of the mitochondria [29–31], respectively, while dynamin-related protein 1 (Drp-1) coordinates fission, along with several accessory proteins, by forming a constricting ring and severing both membranes [32]. Additionally, to maintain turnover of an overall healthy population, new mitochondria are generated via mitochondrial biogenesis while unnecessary or overtly damaged mitochondria are selectively degraded in the process of mitophagy [33].

### mtDNA reduction and clinical relevance

In 2002, Tan et al. analyzed the mitochondrial genome of 19 breast cancer tumor samples and paired normal tissue, finding that 74% had at least one somatic mtDNA mutation [34]. In 2020, Perez-Amado et al. similarly sequenced the mtDNA of 92 paired primary breast tumors and peripheral blood samples, finding somatic mtDNA mutations in 73.9% of the tumors [35]. mtDNA is less protected and more susceptible to damage than nDNA, as it is stored without histones and in close proximity to the generation of reactive oxygen species within mitochondria. Additionally, although mitochondria do have some methods of removing damaged mtDNA [33, 36, 37], they are less effective at removing genotoxic damage than the host cell is [38, 39]. It is not fully known if mitochondrial defects are drivers of tumorigenesis or an effect of the increased proliferation and metabolic demand, although recent mtDNA sequencing and metanalyses indicate that the vast majority, at least, are likely passengers of clonal expansion [35, 40–44]. Furthermore, mutations have only been occasionally observed in areas that may impact OXPHOS or mitochondrial generation, such as a deletion in the conserved OXPHOS polypeptides [44, 45].

Regardless, numerous studies in the last two decades have aimed to quantify overall mtDNA levels and correlate these values to cancer diagnosis and/or patient overall survival prognosis. Interestingly, it appears to be cancer-specific whether mtDNA is increased or decreased in tumorigenic tissue compared to healthy type-matched tissue [46, 47]. The mechanisms of this are not well understood. Most studies report that mtDNA content is lower in breast cancer tissue than in normal mammary epithelium [45, 48, 49]. However, there is also inconsistency across tissue-based studies, wherein some report the lowest mtDNA content in mammary tumors smaller than 2 cm [50], while others report lower mtDNA content in tumors larger than 5 cm compared to smaller tumors [51] or report no trend [52, 53]. Similarly, the clinical effect of decreased mtDNA content in breast cancer is not yet understood and quantifying circulating mtDNA content through blood biopsies has not elucidated a consistent trend in breast cancer [44, 54]. Weertz et al. demonstrated that breast cancer patients in the lowest quartile of mtDNA content (< 350 mtDNA molecules per cell) had a higher probability of metastasis and a shorter distant metastasis-free survival over 10 years. All patients included in this study presented as lymph node-negative and did not receive (neo)adjuvant systemic treatment [50]. Interestingly, Weerts et al. published data from another cohort of breast cancer patients in 2017 where there was no observed correlation between mtDNA content and disease-free survival in patients receiving no adjuvant therapy (24 patients), but, for patients given anthracycline-based adjuvant therapy as part of their treatment (27 patients), higher mtDNA content was associated with lower disease-free survival [55]. This highlights a dramatic issue in analyzing breast cancer patient data—the disparate treatment regiments, which vary due to subtype, geographical region, and across time as new drugs are developed (among other aspects)—it is challenging to create significant sample sizes after this necessary subgrouping, and what differences can be grouped together for a given question is not known.

The mechanism by which altered mtDNA copy number affects breast cancer disease progression is also currently contradictory. Some researchers report that low mtDNA promotes metastasis by inducing epithelial–mesenchymal transition, perhaps via mitochondrial retrograde signaling [40, 56–58], but this is not universally observed [50] and the need for complete mesenchymal transition over phenotypic plasticity and collective metastasis is not fully understood itself [59–62]. Retrograde signaling refers to the pathway of communication from the mitochondria to the nucleus of the cell. It has been speculated that retrograde signaling between nDNA and mtDNA may be responsible for metabolic plasticity/switching during cancer progression [63]. Low mtDNA copy number was also found to generate breast cancer stem cells [40], but this is somewhat contradictory to reports on increased OXPHOS in cancer stem cells [8]. Whether these features are breast cancer subtype specific, model specific, or metastatic location specific is unknown until more research is conducted. Simultaneously, preclinical research is also being conducted analyzing the role of specific mtDNA mutations—often but not limited to the complexes of the ETC—on breast cancer progression, which are beyond the scope of this review.

## Intracellular transportation of mitochondria in breast cancer

### Breast cancer migration

Because the mitochondrial network is not homogeneously distributed throughout the cell, an energy gradient is created. This network of mitochondria is mobile and its distribution varies with the changing metabolic needs of the cell [64]. For example, mitochondria are localized to the leading edge of a migrating cell to provide ATP, given the metabolically demanding nature of migration [30, 64–66]. Specifically, mitochondria are trafficked via Miro1 to supply ATP for lamellipodia formation [30, 64, 66, 67], which can be an important first step in migration as well as for focal adhesion stability [64, 66] and membrane ruffling [64] at the leading edge of the cell. Fibroblasts lacking Miro1 experienced decreased lamellipodia protrusions and impaired actin-based membrane reorganization, resulting in an overall slower migration [64]. Miro1 stands for mitochondrial Rho-GTPase 1 and is the microtubule-based mitochondria transport molecule. The ability of a cell to efficiently shuttle around its mitochondria is dependent on both Miro1 and the structure of the mitochondria itself. Specifically, the mitochondrial network must be capable of breaking apart into smaller mitochondrial segments that may be easily repositioned using fission and fusion as previously discussed. Thus, if the mitochondrial dynamics of a cell are less “pro-fission”, larger mitochondrial networks are observed that are more uniformly distributed throughout the cell due to the increased difficulty in transporting them [30, 65]. Indeed, a similar effect of decreased lamellipodia formation was seen in breast cancer cells (MDA-MB-231, and MDA-MB-436) by altering the expression of fission and fusion molecules, Drp-1 and Mfn1, respectively, wherein “pro-fission” cells (i.e. high Drp-1 and/or decreased Mfn1 expression) with short networks of tubular or spherical mitochondria were more highly migratory [30]. In addition to migration speed, mitochondrial distribution to the leading edge seems to aid other metrics of migration efficiency as well. Specifically, breast cancer cells (MDA-MB-231) with a greater portion of mitochondria in their anterior achieved faster migration velocities, demonstrated greater directional persistence, and more efficiently adapted to alterations in channel confinement (e.g. reduction from larger to smaller channel width), compared to cells with a more symmetric distribution. Interestingly, asymmetric mitochondrial localization does not seem to be required for migration on two-dimensional surfaces that lack chemoattractants or mechanical confinement [65].

It is important to consider that cancer cells can migrate using several different modes: mesenchymal migration, collective movement, or amoeboid migration [68]. Specifically, increased collagen density has been shown in melanoma and fibrosarcoma to trigger the cancer cells to switch from single cell to collective migration to minimize the need for individual proteolytic degradation of collagen and track clearance [69]. Leader–follower dynamics have also been observed in invading breast cancer cells (MDA-MB-231), where a select few cells take on the role of spearheading collective migration through the extracellular matrix [70, 71]. This metabolically demanding task results in a decrease in the ATP/ADP ratio in breast cancer leader cells overtime and prompts more energetic follower cells to replace the leader cells in a relay-like manner to progress invasion. Breast cancer leader cells consume more glucose compared to their follower cells to fuel their role as a leader [70]. However, this may be cancer-type specific, as the opposite metabolic proportions were seen in leader lung cancer cells [72]. Given that breast cancer cells expressing increased Drp-1 exhibited greater migration in vitro [30], breast cancer leader cells may have pro-fission mitochondrial networks that enable them to lead collective migration. Though such a connection between mitochondrial fission and leader cells has yet to be defined for breast cancer, Drp-1 expression is known to be required for the fragmented mitochondrial network of border cells migrating collectively during the development of *Drosophila* [73]. Future efforts will be necessary to elucidate the role of fission in breast cancer leader cells.

### Mitochondria movement in metastasis

Similarly, Drp-1 expression is associated with greater metastatic ability in vivo in breast cancer. Parida et al. isolated latent brain metastases in athymic mice from the HCC1954 and SKBR3 HER2+breast cancer cell lines. These metastatic cells had smaller, more fragmented mitochondria and a greater expression of Drp-1. Intracardial injection of doxycycline-inducible Drp-1 depleted cells (using shRNA against the dynamin 1 like gene (DNM1L) that encodes Drp-1) showed a significant reduction in metastatic lesions for both cell lines [74].

Drp-1 expression appears low in healthy human breast tissue, as characterized via immunostaining. However, Drp-1 staining was observed to be dramatically more intense in samples of invasive carcinoma or samples demonstrating local lymph node metastasis [30]. High Drp-1 expression was also correlated with poor metastasis free survival regarding brain metastases in HER2+breast cancer patients [74]. Interestingly, heightened Drp-1/DNM1L expression in cancerous and metastatic tissue compared to normal tissue does not appear to be breast cancer specific. In many cases, Drp-1/DNM1L was a suggested biomarker postoperatively to predict recurrence [75–78]. Preclinical work has shown that inhibiting Drp-1-dependent fission may reduce or prolong the time until metastatic relapse. For example, beginning 1 week after intracardial injection, oral treatment of mice with mitochondrial division inhibitor 1 (Mdivi-1) (reported to inhibit Drp-1-dependent fission) significantly reduced the number of surviving latent cells and attenuated brain metastasis development [74, 79]. Similarly, Leflunomide, an FDA-approved arthritis drug, was shown to increase Mfn2 expression and suppress tumor growth in a study of pancreatic ductal adenocarcinoma, indicating its potential repurposed used as a chemotherapeutic agent [80].

Despite the advances made on fission and fusion dynamics, the exact mechanism of mitochondria trafficking in breast cancer has yet to be identified. Trafficking mitochondria has been shown to be AMPK-dependent (adenosine monophosphate (AMP)-activated protein kinase) [67] and triggered by PI3K (phosphoinositide 3-kinases) inhibitors [66] in other cancer types and may be applicable to mitochondrial dynamics across cancer types. Interestingly mechanotransduction from interstitial fluid flow activated mitochondrial AMPK in MDA-MB-231 cells, but not in normal MCF10A cells and did not activate AMPK in other subcellular compartments. Inhibition of mitochondrial AMPK blocked flow-induced cell migration, thus reducing cell migration overall [81]. In total, more research is needed to elucidate the role of intracellular mitochondrial trafficking mechanisms during breast cancer metastasis. While not directly related to intracellular mitochondrial movement, it is also critical to remember that metabolic reprogramming of metastatic breast cancer cells is not a monolith, but that disseminated tumor cells undergo tissue-specific adaptation unique to each distant site based on nutrient and oxygen availability, energy requirements, and perhaps the new mechanoenvironment as well [82].

## The flow of mitochondria from surrounding cells in breast cancer

Broadly, this phenomenon involves the movement of either whole mitochondria or mtDNA between cells and can be stimulated via several methods, including cell fusion [83], extracellular vesicles [84–86], tunneling nanotubes (TNTs) [87–90], and through gap junctions [91] (which may be close-ended TNTs). The free release of mitochondria has also been observed in culture medium and human plasma, but it is debated whether they are functional [92, 93]. Cell fusion is the process by which two independent cells partially or fully merge to create a singular cell (permanently or temporarily), thus mixing mitochondria. In vitro, normal (primary cells), neoplastic (MCF10A), and cancerous (MCF7, MDA-MB-231) breast epithelial cells were co-cultured with mesenchymal stem cells (MSCs) whereby fusion began in less than 5 min, and up to 2% of the population consisted of hybrid cells after 72 h [94]. In noncancerous tissue, cardiomyocyte-stem cell fusion has been observed, including the transfer of stem cell mitochondria to cardiomyocytes that facilitated cardiomyocyte reprogramming in the context of regenerative medicine [83]. Although cell–cell fusion has been reported in several cancer types, including breast cancer [94–96], in vivo mitochondrial exchange via fusion is critically understudied.

### Extracellular vesicle transfer

Extracellular vesicles are lipid bound structures secreted by cells for communication, among various other things [97]. Mitochondria have been observed inside of vesicles in several manners. Foremost, mitochondria typically fuse with lysosomes during mitophagy [98, 99], but have been observed inside of extracellular vesicles as a pathway of elimination when lysosomal degradation is compromised [100] and/or when the cell is under high interstitial fluid pressure [101].

Second are mitochondria or mitochondrial material found in extracellular vesicles that appear metabolically functional or facilitate specific intercellular communications (as opposed to a degradation byproduct in the former) [102]. Intact mitochondria have been reported in larger microvesicles (~ 1000 nm) [103–105], while mitochondrial material (e.g. mitochondrial proteins, mtDNA) have been observed in extracellular vesicles more closely resembling exosome sizes (~ 50–200 nm) [103, 106–108]. Extracellular vesicles containing mitochondria or mitochondria-associated proteins have been shown to hasten disease progression in breast cancer in a few studies. For example, when HER2^−^/ER^+^/PR^+^ breast cancer cells (MCF7 and T47D) were exposed to hypoxic culturing conditions, these cells released small extracellular vesicles that, upon uptake by MCF10A cells, resulted in altered mitochondrial dynamics, increased migration, and an increased mesenchymal phenotype in these recipient cells. Interestingly, the key regulator of this response was integrin-linked kinase (ILK) in the extracellular vesicles, which is not well understood, but can activate Akt, a critical mediator of mitochondria trafficking, and which has recently been implicated in blocking retrograde mitochondrial movements. The accumulation of ILK after exposure to hypoxia was specific to extracellular vesicle communication, as ILK levels were not altered in the whole-cell lysates in normoxic versus hypoxic conditions [109]. In another study, breast cancer cells (MDA-MB-231) released extracellular vesicles containing mtDNA. This packaged mtDNA was necessary and sufficient to transfer invasive behavior to other tumor cells [110]. As a final example, breast cancer cells (MDA-MB-231 and BT-549) were made resistant to cisplatin or doxorubicin in vitro or left as chemosensitive. After co-culture of resistant and sensitive cells, bidirectional exchange of mitochondria was observed primarily through direct transfer via extracellular vesicles. After mitochondria exchange (including conditioned media studies rather than co-cultures), chemosensitive triple negative acquired the same chemoresistance [85]. Of note, considerably less work has studied potential effects on the donor cell after losing a portion of mitochondria. For example, here, when “chemoresistant” mitochondria was donated and “chemosensitive” mitochondria was acquired, it was not investigated whether the donor cells maintained the same level of chemoresistance, or if there was temporary or permanent reduction. Although the effect of mitochondrial loss in donor cells is occasionally studied after mitochondrial transfer via tunneling nanotubes (TNTs) (see “Tunneling nanotube (TNT) transfer”), we found no work investigating the same after extracellular vesicle transfer, and donor cell behavior, in general, requires greater study.

Lastly, a subset of vesicles has recently been described which are vesicles that contain mitochondrial material and originate from the mitochondria [102]. Specifically, mitochondrial-derived vesicles are currently categorized as those that deliver mitochondrial content to other organelles, such as lysosomes [111]. In this way, some studies identify mitochondria-derived vesicles as the subpopulation that may handle mitochondria degradation in autophagy-deficient cells, including the BT549 breast cancer cell line [112–114]. In at least some circumstances, the inclusion of mitochondrial content inside of extracellular vesicles appears to be dependent on the formation of mitochondrial-derived vesicles [111, 115], but this phenomenon has not yet been studied enough to draw clear conclusions.

### Tunneling nanotube (TNT) transfer

**Fig. 2:**
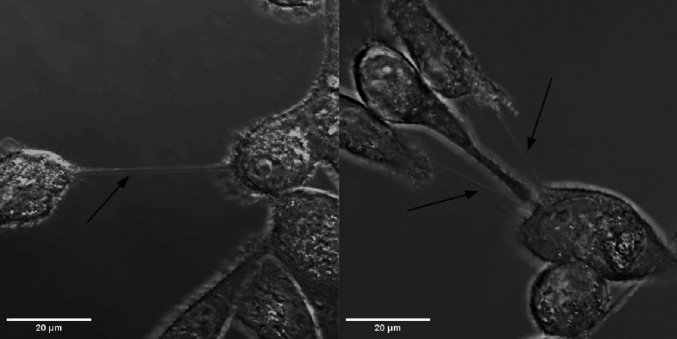
Homotypic TNTs (indicated by arrows) between MDA-MB-231 breast cancer cells in vitro, scale bar 20 μm

The formation of TNTs and subsequent intercellular movement of organelles like mitochondria via TNTs, was not reported until 2004–2006 [116, 117]. TNTs are thin, membrane projections that are suspended above the substratum and bridge independent cells to enable the transfer of cytoplasmic contents (Fig. 2) [116].

**Fig. 3:**
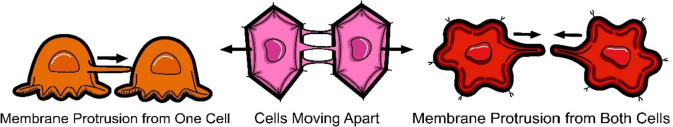
Methods of tunneling nanotube formation

These structures are roughly categorized based on their dimensions and composition. Thin TNTs (< 1 µm in width), composed of only F-actin, span shorter distances, while thick TNTs (> 1 µm), containing F-actin and microtubules that reinforce the structures, span larger distances, even up to and over 500 µm [116, 118, 119]. Cancer cells typically form thin TNTs around 50–200 nm in width and around 30 µm in length, although thick TNTs have been reported in a few studies [119, 120]. TNTs can be formed via several different methods, summarized in Fig. 3. One method is by cell dislodgement whereby the movement of partially or completely attached adjacent cells away from each other draws out nanotubes [87, 121, 122]. These structures can also be formed via connecting membrane protrusions as one or both of the cells’ membranes extend and meet the target membrane [87, 116, 123–125].

Both open-ended and close-ended TNTs have been observed. Open-ended TNTs allow free cytoplasmic transfer, while close-ended TNTs form a junctional border with the target cell, but most TNT literature discusses open-ended TNTs given that it is unclear if close-ended TNTs are simply an intermediate step before full fusion has occurred [120, 126, 127]. In addition, the transfer of material may be unidirectional or bidirectional and often depends on the cell types involved [128, 129]. Also of note, TNTs may form between cells of the same cell type or different cell types, known as homocellular/homotypic and heterocellular/heterotypic TNTs, respectively [87, 123, 125]. Although the current mechanism of TNT inception remains unclear, it is known that inducing stress (e.g. nutrient deprivation, oxidative stress) and damage within the recipient cell can stimulate the formation of TNTs and evoke the transfer of mitochondria [118, 122, 130].

Breast cancer cells have been found to form TNTs with several nearby cell types and subsequently receive mitochondria from endothelial cells [87], cancer-associated fibroblasts (CAFs) [131], MSCs [87], and immune cells [88]. Interestingly, at least one report has shown that the unidirectional transfer of mitochondria to breast cancer cells negatively affected the donor cell (here natural killer T and CD3+/CD8+ T cells). Specifically, after mitochondrial transfer, the immune cells had a significant reduction in basal respiration and spare respiratory capacity as well as significant population loss (cell death) [88]. The effect, especially the effect in vivo, of mitochondrial transfer to/from cancer cells on the non-cancerous populations of the tumor microenvironment is critically understudied.

#### Mesenchymal stem cell (MSC) donors

MSCs are able to differentiate into many different cell types, a property known as multipotency, and are often implicated in regeneration and rejuvenating damaged cells [83, 89, 90, 130]. The accumulation of stressed cells within a tumor similarly stimulates MSCs, causing them to migrate towards the tumor [132] and their nearby presence has been shown to increase the number of breast cancer metastases in mice [133]. mtDNA (and potentially reactive oxygen species) released by injured cells are engulfed by MSCs, which subsequently triggers enhanced mitochondrial biogenesis through retrograde signaling whereafter MSCs have been known to donate their mitochondria to the damaged cells to restore their impaired mitochondrial function via TNTs [89, 90, 130, 134]. Breast cancer cells have been seen forming TNTs with MSCs, as well as transferring mitochondria via this method [87, 135]. Additionally, the artificial transfer of MSC mitochondria to breast cancer cells has been shown to increase their OXPHOS, ATP production, invasion, and proliferation [135], as well as their chemoresistance to cisplatin [136]. The transfer of mitochondria appears to be a selective process as TNTs were formed with breast cancer cells to/from both endothelial cells and MSCs, but in this model, mitochondrial transfer was much more robust from endothelial cells than the MSC TNTs [87]. This may be representative of the composition of the donor cells and/or different mechanisms used by breast cancer cells to stimulate mitochondrial donation from said cells. For example, transfer of mitochondria from MSCs to osteosarcoma cells was shown to be limited to the condition of near total absence of mitochondria function, rather than to replace mtDNA mutations in the cancer cells, but whether this is universal across cell types is not known [137]. Overall, the transfer of mitochondria via TNTs tends to increase OXPHOS and total ATP production, stimulate energy-intensive processes like proliferation and/or migration, and increase chemoresistance of breast cancer cells, as highlighted in Table 1 in “Table of mitochondrial-transfer studies in breast cancer”. It is also important to note, however, that TNT formation itself, without the confirmed transfer of mitochondria, can also propagate chemoresistance [122, 138]. Here, TNTs may be used for bidirectional cytoplasmic exchange where the recipient cell redistributes the chemotherapeutic drug to donor cells. Similar has been seen with microparticles, wherein several chemotherapeutics could be sequestered and removed via microparticles, thus reducing free drug concentration in MCF7 cells [139], but future work is necessary to validate if this compartmentalization and/or transfer of drug occurs via TNTs.

#### Cancer-associated fibroblast (CAF) donors

Mitochondrial transfer via TNTs was also recently identified between CAFs and breast cancer cells [131]. CAFs are an abundant stromal cell type, representing up to 80% of tumor mass in breast cancer, and have been implicated in affecting breast cancer growth and dissemination in several ways [140–142]. Goliwas et al. observed that CAFs increased migration of aggressive breast cancer cell spheroids (MDA-MB-231 and SUM159) through collagen primarily through the formation of TNTs and transfer of mitochondria-containing cargo, as opposed to through other mechanisms, such as reorientation of the collagen fibrils. Allowing the heterocellular TNT formation or artificially transferring CAF mitochondria to the breast cancer cells increased mitochondrial ATP production, for an overall increased total ATP production, in the breast cancer cells [131]. Increased ATP production from artificially transferring CAF mitochondria has been shown to increase the lifetime of breast cancer leader cells, further connecting mitochondria to breast cancer progression [70].

Although the definition of a CAF is debated, including what cell origins may be defined as a CAF rather than being another cancer-associated cell type, endothelial cells can be considered as a source of CAFs in the tumor microenvironment (it is also worth noting that MSCs can potentially be a CAF-origin cell as well) [141, 143, 144]. If extracellular vesicles or mitochondria are isolated from these cell types and given to cancer cells, these would not be considered CAF results since the donor cells did not receive tumor signals. If, however, the cells are placed in a co-culture and allowed to exchange factors/cargo (in via TNTs, extracellular vesicles, soluble factors, etc.) bidirectionally with cancer cells, these results may be more comparable to studies which have first isolated patient- or mouse-derived CAFs as their donor cell before cargo isolation/co-culturing. In general, it is important to recognize the significant influence that tumor cell signaling may have on the cargo released by non-tumor cells, which may be the cause of disparate pro- or anti-tumorigenic effects of said cargo across studies given the varied forms of mitochondrial transfer possible (e.g. co-culture, artificial transfer).

#### Immune cell donors

Normal breast tissue contains a population of immune cells to maintain healthy function, but as breast cancer develops and progresses, there is an increase in the number of infiltrating immune cells [145]. Breast cancer cells have been specifically observed to recruit T cells [146] and macrophages [147] into the tumor microenvironment. These immune cells can then stimulate the formation of homocellular TNTs among breast cancer cells [123, 148] and can also participate in forming heterocellular TNTs with the breast cancer cells [88, 123, 125]. For example, macrophages secrete epidermal growth factor (EGF) and once in close proximity, can stimulate the expression of M-sec and induce homocellular TNT formation in breast cancer cells in a paracrine manner [123, 125, 147]. This process is mechanistically similar to the formation of homocellular TNTs in macrophages [124]. Interestingly, breast cancer cells cultured in macrophage-derived conditioned media formed microplasts, cytoplasmic fragments which contained mitochondria and could fuse with cell membranes [148]. Formation of TNTs between macrophages and breast cancer cells increased the directional migration, invasion, and tumor growth in vivo in zebrafish, although the cargo was not identified to determine if it included mitochondrial content [125]. In fact, mitochondrial transfer between breast cancer cells and macrophages has yet to be visualized, but it is likely given that mitochondria transfer between various T cells and breast cancer has been shown [88]. Termed mitochondrial hijacking, mitochondria from T cells were predominantly trafficked unidirectionally to breast cancer cells, resulting in the oxygen consumption rate, basal respiration, spare respiratory capacity, and proliferation of the breast cancer cells to increase [88]. Given that there is much that remains to be learned about this method of cell-to-cell communication, it is likely that other immune cells and cell types present within the human body will be implicated in mitochondrial transfer via TNTs to breast cancer cells during disease progression.

#### In Vivo models and unknown origin donors

Several groups have developed cancer cells devoid of functional mitochondria (often devoid of mtDNA), typically called ρ^0^ cells, which are then injected into mice. In this method, mitochondrial recovery (via heterocellular transfer) can be observed and the effect on the full range of the metastatic cascade can be studied. However, in these models, it is typically not possible to then confirm the donor cell type or transfer method. Dong et al. observed that injected ρ^0^ melanoma (B16) cells acquired mtDNA through transfer of whole mitochondria and recovered mitochondrial respiration capabilities. Knockdown of mitochondrial complex I and complex II subunits by shRNA in ρ^0^ cells significantly reduced or completely abolished their ability to form tumors, highlighting the role of intact mitochondria on tumorigenesis [149]. Similarly, 4T1ρ^0^ cells were generated and could form tumors in mice due to acquisition of host mouse mtDNA [150]. Interestingly, tumor formation lagged about 20–25 days in 4T1ρ^0^ injections compared to wildtype 4T1 inoculation, demonstrating the potentially significant time needed for mitochondrial transfer in vivo. In a similar vein, 4T1ρ^0^ cells could metastasize to the lungs after primary tumor formation, but did not colonize the lungs if directly injected intravenously. When the lung metastasis cells were isolated and injected in a new cohort, however, no lag time was seen for primary tumor formation after subcutaneous injection, and lung tumor formation was comparable to wildtype 4T1 cells after intravenous injection. The 4T1 cells also recovered more respiratory capability with each stage of the metastatic cascade completed, with the isolated 4T1ρ^0^ lung metastasis cells having recovered to levels not significantly different from wildtype parental 4T1 cells [150]. A follow up study demonstrated that, in addition to bioenergetic remodeling, the transfer of host mitochondria to 4T1ρ^0^ cells in *vivo* is associated with re-expression of genes related to stress adaptation and immune cell recruitment [151].

The methods of mitochondrial transfer, namely TNTs or extracellular vesicles, are certainly not mutually exclusive, although it is unknown what factors influence the use of one versus the other in situations where both are possible [85, 131]. Although TNTs require physical connection, extracellular vesicles may facilitate long-distance mitochondria movement, such as during the development of the premetastatic niche or during communication between primary and secondary tumors [152]. Many studies have demonstrated the important role of extracellular vesicles on metastasis by using intravenous extracellular vesicle injections to prime premetastatic niches prior to intravenous cancer cell injection, which lead to an increase in cancer cell colonization [153, 154]. Unfortunately, analysis of mitochondrial content in extracellular vesicles and, moreover, correlation between mitochondrial EV content and metastatic potential of the source cell line, is lacking and warrants study.

Lastly, recent evidence has shown that neutrophil extracellular traps (NETs) from aged neutrophils in the premetastatic lung niche capture disseminated tumor cells. These NETs were mitochondria-dependent in formation and contained mitochondria [155]. It is not clear if the captured tumor cells take up this mitochondria upon arrival, though another study has shown that colorectal cancer cells treated with NETs increased ATP production, upregulated mitochondrial biogenesis associated genes, and had increased expression of Drp-1 and Mfn2, as well as mitophagy-linked proteins, PINK1 and Parkin [156]. This unique potential transfer method highlights the need for more in vivo experimentation, particularly with a focus on the latter portions of the metastatic cascade.

### Potential clinical utility

In the landscape of mitochondria targeting, current efforts for treatments have focused on artificial mitochondrial transplantation to deliver healthy mitochondria to breast cancer cells and rewire the tumor cells’ mitochondria, potentially leading to increased chemosensitivity and apoptosis [157–159]. More research, and potentially the inclusion of MSCs into the workflow, may be beneficial for optimization given MSC ability to transfer mitochondria to chemotherapy-damaged cells [90]. In addition, new avenues of treatment are being considered, namely combination therapy targeting TNT formation (and/or mitochondrial dynamics), which are showing promise to aid the accumulation of chemotherapeutics within breast cancer cells and increase the treatment efficiency [74, 88, 122]. For example, patients with diabetes receiving the anti-diabetic drug, Metformin, have a lower incidence of cancer as well as a better prognosis if diagnosed with cancer, than patients not receiving metformin [47]. Metformin is known to inhibit TNT formation [131], although it has also been shown to directly disrupt complex I of the ETC in cells [160], so the dominant mechanism is not known [161]. Cytochalasin B has also been shown to inhibit TNT formation. In one study, treatment with cytochalasin B in CAF:breast cancer cell co-culture spheroids inhibited transfer of CAF mitochondria into breast cancer cells and reduced migration speed and overall invasion into the surrounding collagen [131], while another study demonstrated that the chemotherapy agent 5-fluorouracil (5-FU) was more cytotoxic to MCF7 cells when cytochalasin B was given in combination [122]. These studies are early indication of the therapeutic benefit possible with combination TNT-targeting treatments. Some commonly used chemotherapeutic agents, such as Taxanes and Vinca alkaloids, have also been recently shown to partially inhibit mitochondrial transfer by inhibiting microtubule formation, and it may be beneficial to begin using them as adjuvant therapies in a wider range of treatment regiments. This is not a comprehensive list, and several other regulators of TNT formation, such as M-sec, have been proposed for therapeutic purposes, but much more research is needed before clinical benefit may be assessed [88, 159].

It’s important to note that transferring of mitochondria is not the only intercellular interaction that enhances cancer cell mitochondrial respiration. For example, in the Reverse Warburg effect, cancer cells induce aerobic glycolysis in surrounding stromal cells and use the waste metabolites from those cells to undergo additional OXPHOS reactions and fuel cancer growth/invasion [131, 162]. Although treatments targeting certain metabolic pathways or cargo transfer mechanisms seem promising, it has been shown likely from past experience that at least a subset of cancer cells will adapt to a new mechanism to fuel growth afterward. Given that cells from metastatic sites may show different metabolic preferences than the primary tumor cells, if treatments resulted in the inhibition of primary tumor growth at the expense of increasing metastasis to certain sites, this would not be clinically beneficial [5, 163–165]. There is not yet a clear consensus on what metabolic pathways may be more prominent at what metastatic sites (given that it is likely molecular subtype specific and influenced by other patient characteristics), so the effects of these mitochondrial transfer inhibitors cannot be known. Additionally, we do not yet know if the changes in the dominant metabolic pathway across the cell population is due to selection pressures whereby only certain phenotypes present in the primary tumor thrive in certain environments [166], or if the process of metastasizing, including undergoing fluid shear stress, and the features of the new tissue reprogram tumor cells once they arrive, but this difference should certainly affect the development of therapeutics.

## Table of mitochondrial-transfer studies in breast cancer

Table 1 lists studies reporting transfer of mitochondrial content to/from breast cancer cells. It is important to note that there are other reports of mitochondrial transfer between cell types that are not related to breast cancer. This includes other cancer types, other disease states, and in healthy tissue [167–169]. Some of these studies are discussed in the text, but a comprehensive list is beyond the scope of this review. We acknowledge that much of what is seen across other cell types may be applicable to breast cancer, including the array of donor cells possible and the resulting effects of mitochondrial transfer on the recipient cells. However, as briefly noted in “mtDNA reduction and clinical relevance” and “Breast cancer migration”, the metabolic reprogramming necessary for invasion and seen in metastatic populations seems unique to both the cancer cell of origin and the metastatic location. Therefore, it will be important to confirm results in a breast-cancer specific model, as well as noting the molecular subtype.

It is also important to note that there are innumerable additional manuscripts which have reported changes in breast cancer cell behavior, including metabolism shifts, after supplementing with conditioned media and/or extracellular vesicles from another cell population or co-culturing and allowing TNT formation with said population, but which either did not investigate the cargo being transferred or the reported cargo was not directly related to intact mitochondria or mtDNA [123, 125, 170–172]. For example, uptake of extracellular vesicles from Adriamycin-resistant breast cancer cells (MCF7) resulted in increased drug resistance in previously-sensitive MCF7 cells. Chemoresistance was due to delivery of Hsp70 (heat shock protein) via the extracellular vesicles. Delivered Hsp70 translocated into the mitochondria of the recipient cell resulting in impaired mitochondrial respiration and increased glycolysis, but because the cargo itself was not mitochondrial content, this work would not appear in Table 1 [173]. Similarly, several papers identify homotypic TNT formation as a method of aggregation for physical networks of breast cancer cells to develop or as breast cancer clusters and/or spheroids are developing in vitro without discussion of cargo [148, 174, 175]. As above, a few examples of these studies have been included in the text when applicable, but they have been excluded from Table 1. Lastly, there have been reports demonstrating that cancer cells can induce mitochondrial dysfunction/increased glycolytic reliance in cells of the microenvironment via extracellular vesicles and TNTs, including T cells (but not B or natural killer cells) [176], myoblasts (C2C12) [177], and macrophages (polarized to M1) [178], but not specifically related to a known transfer of mitochondria to said cells. These exclusions demonstrate the plethora of work needed to expand our understanding of the bidirectional effects between the tumor and its microenvironment in breast cancer.

**Table 1:** Intercellular movement of mitochondria in breast cancer

Donor cell	Recipient cell	Transfer method	Recipient effect	Citation
Endothelial cells (E4ORF1+)	MCF7 and MDA-MB-231 breast cancer cells	TNTs	After co-culture, MCF7 cells were sorted into those that had received endothelial mitochondria and those that had not. Mitochondrial recipient cells displayed the greatest chemoresistance to doxorubicin MSC co-cultures were also investigated. Breast cancer cells formed TNTs with MSCs, but mitochondrial transfer was not robustly seen in this study	[87]
Platelets	MDA-MB-231 and MCF7 breast cancer cells. MCF10A mammary epithelial cells	Microparticles/extracellular vesicles	Mitochondrial-containing microparticles were taken up by MDA-MB-231 and MCF10A cells, but not MCF7. MDA-MB-231 cells, being the most permissive, had a significant increase in basal respiration and total ATP (majorly from OXPHOS) after platelet microparticle uptake, as well as increased migration through a Matrigel-coated porous membrane	[179]
			Interestingly, a similar study used platelet-derived extracellular vesicles and did not find an increase in mitochondrial function after uptake in MDA-MB-231 or BT474 cells, although there was a significant increase in invasion of MDA-MB-231 cells	[180]
CAFs	MDA-MB-231 and SUM159 breast cancer cells	TNTs	Mitochondria from CAFs increased migration through collagen in breast cancer spheroid models. Breast cancer cells had increased mitochondrial ATP production with negligible impact of glycolytic ATP production after mitochondrial transfer	[131]
MDA-MB-231 cells resistant to cisplatin or doxorubicin	Parental MDA-MB-231 cells (chemosensitive)	Extracellular vesicles	Culturing parental cells in conditioned media or with extracellular vesicles from chemoresistant variants lead to acquired resistance to the same chemotherapeutic. Mitochondrial transfer was observed and mtDN4 mutation acquisition was required Acquired chemoresistance was also seen in BT-549 cells when using conditioned media from chemoresistant BT-549 cells on parental BT-549 cells. The effect was smaller than in MDA-MB-231 cells and were not used in additional studies	[85]
T Cells, including Natural Killer, CD3+/CD8+, primary isolated T cells	MDA-MB-231 and 4T1 breast cancer cells	TNTs	Mitochondrial transfer metabolically empowered breast cancer cells (i.e. higher basal respiration and spare respiratory capacity) and depleted immune cells. Breast cancer cells had significantly higher proliferation rates due to mitochondrial transfer, and immune cells had a reduction in cell population after transfer	[88]
MDA-MB-453 cells treated with ROCK-mTOR inhibitor (fat-like cells)	Untreated MDA-MB-453 breast cancer cells	TNTs/A microfluidic device that mimicked TNTs before artificial transfer	After traveling through the microfluidic device, mitochondrial released endonuclease G into recipient cells (named unsealed mitochondria). Unsealed mitochondria did not induce apoptosis, but the recipient cells were more chemosensitive to doxorubicin and induced apoptosis in response to caspase-3. Doxorubicin experiments were repeated with MDA-MB-231 and MCF7 breast cells with similar results. In vivo, a low dose of doxorubicin with unsealed mitochondria inhibited tumor growth which was not seen in doxorubicin-only control mice	[181]
Murine CAFs isolated from xenografts	MCF7, ZR751, T47D, and BT474 breast cancer cells (after hormone therapy)	Extracellular vesicles	CAF extracellular vesicles housed the entire mitochondrial genome. mtDNA transfer via extracellular vesicles rescued OXPHOS capabilities in breast cancer cells. This promoted an escape from metabolically-induced dormancy/quiescence in breast cancer cells	[182]
MDA-MB-231 breast cancer cells	Glutamine-starved MDA-MB-231 cells	Extracellular vesicles	Extracellular vesicles from parental MDA-MB-231 cells enabled glutamine-starved recipient cells to degrade collagen and invade into the surrounding extracellular matrix. If mGluR3 was inhibited or PINK1 was knocked down in the donor cell line, the extracellular vesicles did not enable recipient cell invasion. Through the generation of an mtDNA deficient donor cell line, the acquisition of the invasive phenotype was seen to be dependent on the packaging of mtDNA into vesicles by the donor cells. This was confirmed through the creation of liposomes with mtDNA as a surrogate for the donor extracellular vesicles	[110]
MCF7 and T47D breast cancer cells (maintained in hypoxia)	MCF10A normal mammary epithelial cells	Extracellular vesicles	MCF10A cells showed increased migration and larger velocity in several assays, owing to more dynamic intracellular mitochondrial movement. Extracelluar vesicles from hypoxia-induced breast cancer cells also stimulated focal adhesion turnover in MCF10A cells. RNA sequencing additionally showed increased pro-inflammatory responses, increased markers of epithelial-mesenchymal transition, and reduced cell death responses. These changes were not or were only minimally induced if given breast cancer cells cultured in normoxic conditions	[109]
			In a follow-up study, extracellular vesicles from hypoxia-induced breast cancer cells MCF10A cells were also shown to stimulate mitochondria dynamics, with increased rates of both fusion and fission, and alter mitochondrial localization in MCF10A cells	[183]
P-glycoprotein oxer-expressing drug resistant MCF-7 variants	Parental (drug sensitive) MCF-7 cells	Microparticles and TNTs	Functional P-glycoprotein was transferred to parental MCF7 cells. Membrane P-glycoprotein overexpression is connected to multidrug resistance through an energy-dependent drug efflux pump and functionally active P-glycoprotein is expressed in the mitochondrial membrane [184, 185]. Although not identified as mitochondria in the study, organelle transfer was observed in the TNTs	[138]
MDA-MB-231 cells treated with or without green tea polyphenol epigallocatechin-3-gallate (EGCG)	MSCs	Extracellular vesicles	MSCs treated with extracellular vesicles from control (untreated) MDA-MB-231 cells had increased migration compared to untreated MSCs. EGCG treatment reduced the amount of mitochondrial content in isolated extracellular vesicles and reduced vesicle fusion capacity	[186]
MSCs	MDA-MB-231 (and TS/A-p/c) breast cancer cells	TNTs and artificial transfer	Mitochondrial transfer from MSCs was seen for both MDA-MB-231 and TS/A-pc cells. Artificial transfer of MSC mitochondria was performed on MDA-MB-231 cells whereafter increased ATP production, a higher oxygen consumption rate, and decreased glycolytic activity were observed. After transfer, the cancer cells also had increased migration and invasion. Interestingly, these functional effects were present when roughly 1.25 µg of MSC mitochondria were present, but diminished if roughly 5 µg or more was transferred	[135]

Papers published in or after 2011

## A mito-centric perspective in the future

Mitochondria have fascinated researchers for decades, and the recent discovery of tunneling nanotubes has added an additional element to consider for the role of mitochondrial transfer in cells. Focusing on mitochondrial dynamics and transfer via TNTs may yield new therapeutic targets and combination treatments for breast cancer patients. Examining the ways in which chemotherapeutics interact with the mitochondria of breast cancer cells could become standard practice in drug development, as some chemotherapeutics can stimulate the redistribution of mitochondria to the anterior portion of the cell to promote tumor invasion [66]. Additionally, there are several groups investigating the use of extracellular vesicles/nanoparticles targeting mitochondria/mitochondrial damage in order to induce cell death in breast cancer cells or achieve M1 macrophage polarization and macrophage tumor infiltration [187–192]. With regard to retrograde signaling, and given that mtDNA is more susceptible to mutations than nDNA, how mitochondria interact with oncogenes and tumor suppressors may be of utmost important and its investigation could uncover new upstream, therapeutic targets. How mitochondria sense their intracellular environment and the cell’s extracellular environment could further help elucidate the mechanics of communication between the mitochondria and host cell’s nucleus. While much of these discussions were beyond the scope of this review, they represent potential therapeutic options that show promise and overall, we hope to have highlighted the need for more (pre)clinical research on mitochondrial dysfunction and mitochondrial transfer across cancer progression and treatment regimens.

Using mitochondria as a prognostic factor for treatment or predicting overall patient survival will hopefully prove useful to incorporate into analyzing biopsies. As discussed, there are currently many contradictory opinions about the value of identifying mutations in mitochondria, especially since mitochondrial mutations are accumulated with age, but given the reliance of cancer cells on their mitochondria, the genetic composition of mitochondria could be used to predict next steps for patients. Rather than analyzing mtDNA levels from tissue biopsies or cell-free in the plasma, recent studies have highlighted the potential to analyze mtDNA or mitochondrial proteins housed inside extracellular vesicles during liquid biopsies [107, 182, 193]. For example, ND1, which encodes for an enzyme involved in complex 1 in the ETC, was elevated in 86% of patients with breast cancer [182]. Breast cancer extracellular vesicles were also higher for two inner membrane proteins (MT-CO2 and COX6c—mitochondrial encoded cytochrome c oxidase II and cytochrome c oxidase subunit 6c, respectively) [107] and had a loss of Mfn2 and SH3GL2 (SH3 domain containing GRB2 like 2) as opposed to healthy controls [194]. The consensus of a mitochondria-related gene signature in serum-harvested extracellular vesicles that correlate to better or poor prognoses would be necessary for these methods to see clinical utilization. Additionally, the different molecular subtypes of breast cancer underpin all discussion of clinical utility as these differences are almost certainly critical for prognostic evaluations and effective response to treatments.

Besides identifying the role of mitochondria within cells, mitochondrial transfer via tunneling nanotubes brings the opportunity to further uncover the role of TNTs in cell–cell communication. Since 2004, many groups have begun to identify TNTs within their own research, yet few papers have examined the events before or after mitochondrial transfer. There are still questions surrounding how cells stimulate the formation of TNTs, what dictates unidirectional versus bidirectional transport of TNT cargo, and what establishes the donor-recipient cell hierarchy. In cells that form TNTs as a mechanism to aid damaged cells, how is the need interpreted and is there a threshold where TNTs will not form if the cellular damage is deemed beyond repair? Even after documenting mitochondrial transfer via TNTs, questions about the functionality of the transferred mitochondria, incorporation and downstream implications to the recipient cell, and the effect of mitochondrial loss on the donor cell remain. Though mitochondria have been identified as TNT cargo, it still remains unclear what else may be transferred and how cells select what to transfer to recipient cells. Future efforts will be necessary to understand what kind of cell-to-cell signaling is required to stimulate the donation of such a precious and valuable component, as the mitochondrion.
